# Automated FingerPrint Background removal: FPB

**DOI:** 10.1186/1471-2105-10-127

**Published:** 2009-04-30

**Authors:** Simone Scalabrin, Michele Morgante, Alberto Policriti

**Affiliations:** 1 Istituto di Genomica Applicata (IGA), via J. Linussio 51, I-33100 Udine, Italy; 2 Department of Mathematics and Computer Science, University of Udine, via delle Scienze 206, I-33100 Udine, Italy; 3 Department of Agriculture and Environmental Sciences, University of Udine, via delle Scienze 208, I-33100 Udine, Italy

## Abstract

**Background:**

The construction of a whole-genome physical map has been an essential component of numerous genome projects initiated since the inception of the Human Genome Project. Its usefulness has been proved for whole-genome shotgun projects as a post-assembly validation and recently it has also been used in the assembly step to constrain on BACs positions. Fingerprinting is usually the method of choice for construction of physical maps. A clone fingerprint is composed of true peaks representing real fragments and background peaks, mainly composed of *E. coli *genomic DNA, partial digestions, star activity by-products, and machine background. High-throughput fingerprinting leads to the production of thousands of BAC clone fingerprints per day. That is why background peaks removal has become an important issue and needs to be automatized, especially in capillary electrophoresis based fingerprints.

**Results:**

At the moment, the only tools available for such a task are *GenoProfiler *and its descendant *FPMiner*. The large variation in the quality of fingerprints that is usually present in large fingerprinting projects represents a major difficulty in the correct removal of background peaks that has only been partially addressed by the methods so far adopted that all require a long manual optimization of parameters. Thus, we implemented a new data-independent tool, *FPB *(FingerPrint Background removal), suitable for large scale projects as well as mapping of few clones.

**Conclusion:**

*FPB *is freely available at . *FPB *was used to remove the background from all fingerprints of three grapevine physical map projects. The first project consists of about 50,000 fingerprints, the second one consists of about 70,000 fingerprints, and the third one consists of about 45,000 fingerprints. In all cases a successful assembly was built.

## 1 Background

The construction of a whole-genome physical map [1-6] has been an essential component of numerous genome projects initiated since the inception of the Human Genome Project [7]. Its usefulness has been proved for whole-genome shotgun projects as a post-assembly validation and recently it has also been used in the assembly step to constrain on BACs positions [8].

High-throughput fingerprinting can produce thousands of BAC clone fingerprints per day. Hence automatic editing of corresponding files can be extremely useful.

From now on the description of the process will be based on fingerprints produced on Applied Biosystems Instruments (ABI) automated DNA sequencers, but the method can be applied to any kind of fingerprint as long as similar input is provided. Moreover, the following terms will be used interchangeably since they correspond to the same entity: fragment, band, and peak.

**Figure 1:**
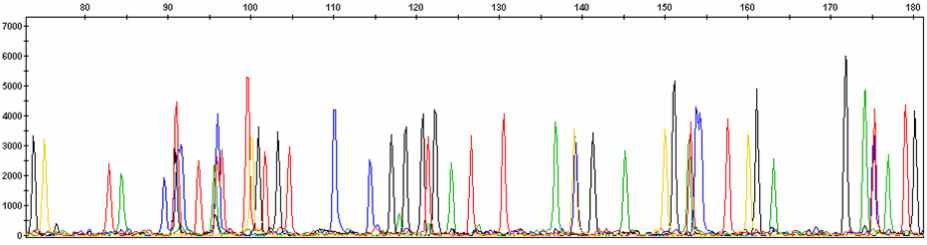
**Example of an electrochromatogram**. *x *and *y *axes represent, respectively, fragment size and peak intensity. Background is composed of low signal peaks (in this example with intensity lower than 500).

Fingerprint data is stored in electrochromatograms, *i.e*. fsa files, output by GeneMapper, ABI sequencer software. Each peak represents a fragment with a certain size and intensity (fig. 1) and it can derive from different sources (see Additional file 1 for details and examples):

• "true peak" derived from a DNA insert digested band;

• low signal peak produced by the machine;

• partial digestion related peak;

• star activity by-product, see [9];

• *E. coli *genomic DNA band;

• vector band;

• out of size standard range band (with unreliable sizing);

• wide area peak (unreliable, resulting from co-migrating fragments).

Last three kinds of peaks can be removed in a preprocessing step. While, low signal peaks produced by the machine, partial digestion related peaks, star activity by-products, and *E. coli *genomic DNA bands can be considered as background signal that needs to be subsequently removed to allow a correct assembly by FPC [10,11], nowadays the only physical map assembler. The vast majority of background peaks is usually related to *E. coli *genomic DNA bands; they tend to exhibit a lower signal than true peaks and therefore they can be removed by computing a threshold below which data needs to be rejected (including low signal peaks produced by the machine). Partial digestion related peaks and star activity by-products tend to produce similar patterns with intermediate signal peaks that, again, can be removed with the use of a threshold on intensity of the signal.

## 2 Implementation

### 2.1 Data

ABI-produced electrochromatograms basically code for "Dye-Sample Peak" (dye and fragment number), "Sample File Name" (clone name), "Size" (fragment length), "Height" (peak intensity), and "Area" (peak's area).

The approach of *FPB *to remove background includes a preprocessing step where fragments out of size standard range, vector bands, and wide area peaks are removed. Then, to achieve the final goal of a correct map assembly, *FPB *follows the following principle:

#### Safety principle

It is better to exclude a few true peaks than to include lots of background.

Such a principle sacrifices sensitivity in favor of specificity for two reasons: first, low sensitivity is counter-balanced by the level of physical coverage needed for a successful physical map, second, high specificity is needed for a correct assembly (remember the example above on vector bands).

**Figure 2:**
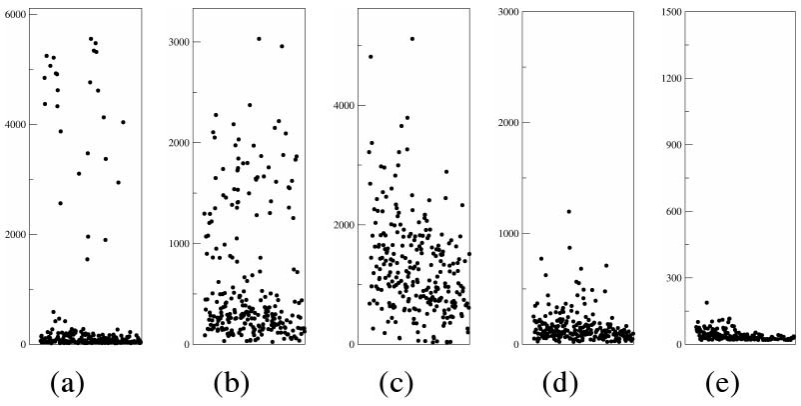
**Alternative representation of peak heights**. Different scenarios: from fingerprints with a clear gap between true peaks and background to only background peaks (empty clones) through intermediate cases.

The major bottleneck in fingerprinting background removal is represented by BAC-DNA quality: from clone to clone the number of distinguishable true peaks can vary highly. In general, the intensity of true peaks is much higher than that of background peaks, consequently our task may be reduced to the determination of a *threshold *below which peaks can be rejected. Unfortunately, for a number of different reasons, lots of scenarios can arise:

• clear fingerprints with an evident *gap *between true and background peaks where to put the threshold, fig. 2(a);

• fingerprints with some low signal true peaks, with no evident gap, fig. 2(b). In this case it is reasonable to remove most, if not all, of the background peaks preserving the most reliable true peaks only, in accordance with the safety principle;

• fingerprints with high signal background peaks, fig. 2(c), very low signal true peaks, fig. 2(d), or without true peaks (empty fingerprints), fig. 2(e). A high threshold should be set or all fragments should be rejected;

• fingerprints with partially digested fragments or by-products of star activity. Such fragments tend to exhibit an intermediate signal intensity, between true and background signal peaks. In accordance with the safety principle they should be rejected.

See table 1 and Additional file 2 for a distribution of possible scenarios occurring in a single project.

**Table 1:** Distribution of fingerprint scenarios

**%**	**a**	**b**	**c**
**Blue**	0.16	0.80	0.03
**Green**	0.62	0.33	0.04
**Yellow**	0.48	0.47	0.04
**Red**	0.81	0.17	0.02

Distribution of different scenarios of fig. 2 in a set of 82,176 fingerprints produced at IGA, Udine, Italy, inside the TriticeaeGenome Project. Clones rejected are 1,207 (1%) and correspond to scenarios d and e. Remaining clones are divided into scenarios a, b, and c for each single dye. It is striking the variability between scenarios a and b and different dyes. See Additional file 2 for details.

At present most of physical mapping projects use fluorescent digestion with SNaPshot method [12] obtaining fragments with four possible dyes and the size standard internal marker. Obviously, the process of background removal has to be iterated for each different dye. *FPB *has directly been tested on ABI 3730 Genetic Analyzer data exported by Genemapper to tabulated text files from original fsa files. Nevertheless, the method can be applied to different kind of processes, *e.g*. single dye on agarose gel fingerprints, as long as similar input is provided.

### 2.2 State of the art and novel method

A few solutions to the problem of removing background from fingerprints have already been proposed. In particular, the first tool used for agarose-gel based fingerprints was Image [13], suitable for small projects since high human interaction is required for true bands determination. A more automated alternative to work on agarose-gel based fingerprints has recently been developed [14]. Unfortunately, such fingerprints suffer of a deep problem: the inaccuracy of bands sizing, *i.e*. a single band can be sized even a few bp apart from its real value. In truth, also capillary electrophoresis based fingerprints slightly suffer of inaccurate band sizing but in a consistent way.

At the moment, the only tools available to remove background from capillary electrophoresis based fingerprints are *GenoProfiler *[15,16] and its descendant *FPMiner *[17]. Unfortunately, the algorithm used in the main background removal step requires a fine tuning by the user of empirically determined parameters (see first method below for details); such a task becomes very hard when analyzing thousands of capillary electrophoresis based fingerprints that may be highly variable in signal intensity.

A similar context in which background removal was successfully applied to is "component detection in liquid chromatography/mass spectrometry" [18,19]. In this case, the use of standard background techniques was of limited success [19]. But in [18] the authors propose to use, not only the true signal, but also the background signal to discriminate between the two signals, and they also propose to refine such discrimination in a step-wise manner.

Next, the description of three methods to remove background after the preprocessing step are presented. The first method is the one used in *GenoProfiler*, while the second and the third approaches exploit similar ideas as in [18] to refine the discrimination of the two signals.

#### First method

The first method, initially implemented in the lab of DuPont Crop Genetics Research and then coded into *GenoProfiler *[16] relies on the assumption that the threshold is linearly related to the intensity of few true peaks.

After sorting all peaks of a dye of a clone in descending order by height, the problem of determining the corresponding threshold can be converted to finding the index of the lowest putative true peak or, if an overlap of true and background peaks occur, finding an index *k *such that the set of the *k *highest peaks contains as many true peaks as possible and as few background peaks as possible. In practice, the first method considers only a few putative true peaks, let us say from the *i*-th to the *j*-th (first i-1 peaks are not taken into account since they can be considered artifacts or not reliable data, *e.g*. dye blobs), computes the average of their heights to establish, to a certain extent, how high is the signal of true peaks, and multiplies such a value by a constant factor, *e.g*. 0.35, previously empirically determined library per library, dye per dye (if fluorescence was used); the value obtained is considered the threshold. This is done for each dye of each clone. At this point, apart from the first *i*-1, only peaks above the threshold are considered true ones. Obviously this approach is quite limited: first of all the determination of constants of multiplication has to be done empirically for each dye and for each library and it relies on the assumption that the signal is highly consistent among different clones in a library. Then, to compute the mean value, it relies only on few peaks without caring of data variability.

GenoProfiler codes for another method to remove background, based on the frequency of peak heights; unfortunately, such a method does not have general applicability: cases as that in fig. 2(c) cannot be solved correctly (we also tried to approximate the distribution of peak heights with curves but with limited success because data variability present in the different scenarios does not respect a fixed distribution; in particular lowest peaks of background are highly abundant and their distribution could be approximated with a gaussian curve while the distribution of higher background peaks could not be distinguished from that of true peaks; an example of unsuccessful application of such a method is directly given by the black line in fig. 1 of the GenoProfiler paper [16]).

Hence the large variation in the quality of fingerprints that is usually present in large fingerprinting projects represents a major difficulty in the correct removal of background peaks that has only been partially addressed by the methods so far adopted that all require a long manual optimization of parameters.

### Second method

A few definitions are introduced to help the reader in understanding this method.

***Definition***. *high limit (hl)*, is a value above which peaks are considered to be real one (apart from artifacts).

***Definition***. *high average (ha)*, is the average value of peak heights greater than *hl *(apart from artifacts).

***Definition***. *low limit (ll)*, is a value below which peaks are considered to be background.

***Definition***. *low average (la)*, is the average value of peak heights lower than *ll*.

**Figure 3:**
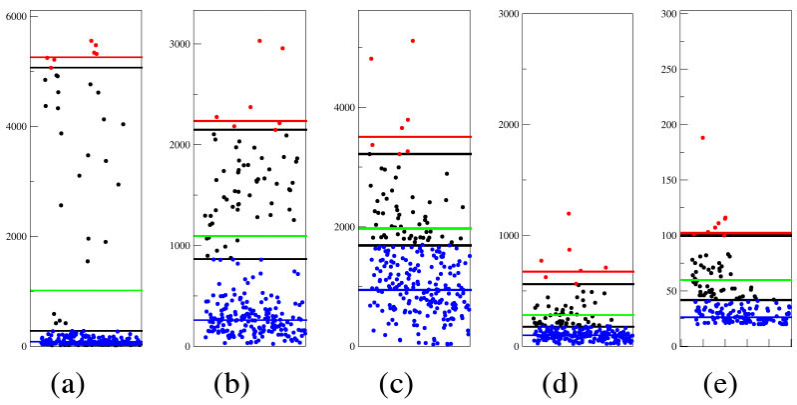
**Application of the second method on the cases of fig. 2**. Red and blue dots are, respectively, above and below *hl *and *ll *(marked by a black line). Herein the first two peaks are considered artifacts and are ignored in the computation of *ha*. Furthermore, only five peaks are used to determine initial *ha *(red line) and peaks below the 60-th are supposed to be background and used to compute *la *(blue line). Notice the variety of cases according to the ratio between *ha *and *la*. The green line represents a tentative threshold based on the ratio between *ha *and *la*.

The second method considers more peaks. It still sorts all peaks of a dye of a clone in descending order by height, then it computes *ha *and *la *based respectively on given *hl *and *ll*. *hl *usually corresponds to the height of the *j*-th peak of the first method while *ll *corresponds to a height below which it can be assured there are no true peaks, *e.g*. if each dye is supposed to have at most *n *true peaks then *ll *can be assumed to be the height of the (*n*+1)-st peak. Comparing the two values obtained for *ha *and *la *and aware of the safety principle, it is possible to forecast a model for true and background peaks: smaller is the *ratio *of *ha *and *la*, closer to *hl *must be the threshold. A graphical representation of this method is presented in fig. 3. In the case *n *peaks are not available then *ll *is set to the height of the 5-th lowest peak, considering the last 5 peaks background. In general, if the ratio between *ha *and *la *is low, as in fig. 2(e), then the clone is rejected.

A big problem of this approach stands in the difficulty of finding a good function to convert a ratio to a threshold. Moreover, another problem is the consideration of only few peaks on the true side, *i.e*. not enough data is considered to determine a reliable high average and consequently the threshold cannot be accurate.

### Third method

Herein the second approach is iterated to consider as many peaks as possible. At each iteration, possibly, more high and low signal peaks are considered and a new ratio is computed. Again, a low number of true peaks is initially considered and at later iterations, if more true peaks are available, it is increased. Before giving the details of the final algorithm, two more definitions are introduced.

***Definition***. *initial gap (ig)*, is the difference between *ha *and *la *as they are computed in the second method. *ig *= *ha*_1_-*la*_1_, where indexes represent the current iteration.

***Definition***. At the *i*-th iterative step, *displacement *is a portion of the difference between *hl*_*i *_and *ll*_*i*_, the difference between *ha*_*i *_and *la*_*i*_, and the *initial gap*. It is used to decrease *hl*_*i *_and partially to increase *ll*_*i *_at each iteration (background tends to vary less than true peaks) to allow the computation of *hl*_*i*+1 _and *ll*_*i*+1_, next limits true and background peaks.

In the implementation of *FPB *the following formulas are used:



suitable for a fast convergence of the iterative process and reliable enough on splitting correctly true and background data at each iteration (data not shown, based on empirical tests). The iteration stops when a gap is reached (no more peaks are available) or when the two limits meet. In both cases, the threshold is set to the value of *hl *after the last iteration. It has to be noticed that the 20%*ig *contribute of the displacement constrains the maximum number of loop iterations to be five.

#### Implementation

We developed *FPB, FingerPrint Background removal*, a Perl script with a Perl/Tk GUI, which codes for the third method. The program also converts data to suit FPC input format. The use of GeneMapper exported tables provides accurate peak sizing. The program is freely available at  along with some additional scripts to visualize data on a Linux platform.

## 3 Results and Discussion

*FPB *can work with any fragment length and on exported tables from both agarose-gel and capillary electrophoresis projects. Moreover, the program does not need long and manual curations to detect optimized parameters, as stated by the authors of the maize HICF map which relied only on the DuPont method to remove background: "after considerable experimentation with parameters" they were able to automatically define thresholds [20].

It has also been tested on a set of random clones from the grapevine physical map project giving the results reported in table 2. *FPB *was able to recognize clones from all three expected categories of clones: chloroplast, centromere, and rDNA, while *GenoProfiler *was partially able to distinguish two of them (chloroplast and centromere) only in the case of manually optimized parameters with the main intent of keeping contigs separated. Resulting contigs were easily veriafiable since BAC-end sequences were available. It is worthwhile noting that all inserts, including chloroplast, centromere, and rDNA, are transformed in *E. coli*, and contain the main background contaminant, the *E. coli *bands. If background removal is including background peaks then non overlapping clones may be incorrectly clustered together, as in the Genoprofiler1 assembly of table 2. Vice versa if the choice of the threshold is too conservative then there is no sufficient information to cluster overlapping clones, as in the Genoprofiler2 assembly of table 2. Instead, neither of the two cases was produced by using *FPB*.

**Table 2:** *FPB *and *GenoProfiler *background removal

**Method**	**Ctg**	**Clones**	**Qs**	**Score**	**Remark**
**FPB**	1	22	2	0.784	Chloroplast
	2	28	16	0.515	Centromere
	3	6	1	0.780	rDNA

**GenoProfiler1**	1	150	105	0.420	Wrong assembly

**GenoProfiler2**	1	22	5	0.767	Chloroplast
	2	22	12	0.556	Centromere

Comparison of contigs produced by FPC (tolerance 0.4 bp, cutoff 1e-20) on a set of 1000 clones of the grapevine physical map project (about 0.2× coverage) and processed with *FPB *and *GenoProfiler*. Clones were chosen at random from a set of 30,000 clones for which BAC-end sequences were available. Only contigs with at least 3 clones are presented in the table. For *GenoProfiler*, we used both default parameters and manually optimized parameters, respectively in methods *GenoProfiler1 *and *GenoProfiler2*. Qs, represent the so-called "questionable clones" and score is a probabilistic value reflecting the goodness of contigs in FPC. The last column of the table specifies which kind of clones compose the corresponding contig (from an independent inspection on the BAC-end sequences of the set of selected clones). FPC produced wrong or incomplete assemblies with, respectively, default or manually optimized parameters for *GenoProfiler*.

Finally, *FPB *was used to remove background from all fingerprints of three different grapevine physical map projects. The three projects, [21,22] and Scalabrin *et al*. (submitted to BMC Genomics), consisted of about 45,000, 70,000, and 50,000 fingerprints respectively. In all cases a successful assembly was built (iterative assembly starting with cutoff 1e-50 decreasing stringency up to 1e-20 through DQ and merge steps): only 2982 (6.63%), 1295 (1.85%), and 2,310 (4.62%) Q clones ("Questionables") were produced by the assembly program, meaning that background was properly removed. Moreover, consider that in two of the maps, [21] and Scalabrin *et al*. (submitted to BMC Genomics) the number of Q clones is highly affected by the heterozygosity of the two selected lines (*Pinot Noir *and *Cabernet Sauvignon*): as demonstrated in Scalabrin *et al*. (submitted to BMC Genomics) FPC assemblies affected by heterozygosity exhibit incorrect positioning of clones in a single contig and therefore lots of "Questionable" clones are produced. In contrast, the assembly of line PN40024, close to full homozygosity, included very few Q clones.

## Conclusion

FPB is freely available at . It requires *Perl *and *Perl/Tk*. *FPB *is effective at automatically removing background in small projects as well as in big projects as demonstrated on the three independent assemblies on different strains of grapevine.

## Authors' contributions

MM proposed the problem and approved the computational solution. SS proposed the main solution, implemented the tool, and verified its efficacy on different maps and datasets. AP improved the solution by proposing the iterative approach and coordinated the computational part of the experiments. All authors read and approved the final manuscript.

## Supplementary Material

Additional file 1 **Particular cases of peaks to be removed**. Partially digested fragments, star activity by-products, "machine" background, and *E. coli *peaks present particular features and need to be removed accordingly. Click here for file

Additional file 2 **Distribution of true peaks vs background peaks**. True and background peaks distribute differently from clone to clone and from dye to dye. Therefore, different scenarios may arise and particular care in background removal should be used. Click here for file

## References

[B1] Gonzalez J, Nefedov M, Bosdet I, Casals F, Calvete O, Delprat A, Shin H, Chiu R, Mathewson C, Wye N, Hoskins R, Schein J, de Jong P, Ruiz A (2005). A BAC-based physical map of the Drosophila buzzatii genome. Genome Research.

[B2] HGP (2001). A physical map of the human genome. Nature.

[B3] Kelleher C, Chiu R, Shin H, Bosdet I, Krzywinski M, Fjell C, Wilkin J, Yin T, DiFazio S, Ali J, Asano J, Chan S, Cloutier A, Girn N, Leach S, Lee D, Mathewson C, Olson T, O'connor K, Prabhu A, Smailus D, Stott J, Tsai M, Wye N, Yang G, Zhuang J, Holt R, Putnam N, Vrebalov J, Giovannoni J, Grimwood J, Schmutz J, Rokhsar D, Jones S, Marra M, Tuskan G, Bohlmann J, Ellis B, Ritland K, Douglas C, Schein J (2007). A physical map of the highly heterozygous Populus genome: integration with the genome sequence and genetic map and analysis of haplotype variation. The Plant Journal.

[B4] Meyers B, Scalabrin S, Morgante M (2004). Mapping and sequencing complex genomes: let's get physical!. Nature Reviews Genetics.

[B5] Ren C, Lee M, Yan B, Ding K, Cox B, Romanov M, Price J, Dodgson J, Zhang H (2003). A BAC-based physical map of the chicken genome. Genome Research.

[B6] Wu C, Sun S, Nimmakayala P, Santos F, Meksem K, Springman R, Ding K, Lightfoot D, Zhang H (2004). A BAC-and BIBAC-based physical map of the soybean genome. Genome Research.

[B7] HGP (2001). Initial sequencing and analysis of the human genome. Nature.

[B8] Warren R, Varabei D, Platt D, Huang X, Messina D, Yang SP, Kronstad J, Krzywinski M, Warren W, Wallis J, Hillier L, Chinwalla A, Schein J, Siddiqui A, Marra M, Wilson R, Jones S (2006). Physical map-assisted whole-genome shotgun sequence assemblies. Genome Research.

[B9] Wei H, Therrien C, Blanchard A, Guan S, Zhu Z (2008). The Fidelity Index provides a systematic quantitation of star activity of DNA restriction endonucleases. Nucleic Acids Research.

[B10] Soderlund C, Humphrey S, Dunhum A, French L (2000). Contigs built with fingerprints, markers and FPC V4.7. Genome Research.

[B11] Soderlund C, Longden I, Mott R (1997). FPC: a system for building contigs from restriction fingerprinted clones. CABIOS.

[B12] Luo M, Thomas C, You F, Hsiao J, Ouyang S, Buell C, Malandro M, McGuire P, Anderson O, Dvorak J (2003). High-throughput fingerprinting of bacterial artificial chromosomes using the SNaPshot labeling kit and sizing of restriction fragments by capillary electrophoresis. Genomics.

[B13] Sulston J, Mallett F, Staden R, Durbin R, Horsnell T, Coulson A (1988). Software for genome mapping by fingerprinting techniques. CABIOS.

[B14] Fuhrmann D, Krzywinski M, Chiu R, Saeedi P, Schein J, Bosdet I, Chinwalla A, Hillier L, Waterston R, McPherson J, Jones S, Marra M (2003). Software for automated analysis of DNA fingerprinting gels. Genome Research.

[B15] You F, Luo M (2005). GenoProfiler User's Manual.

[B16] You F, Luo M, Gu Y, Lazo G, Deal K, Dvorak J, Anderson O (2007). GenoProfiler: batch processing of high throughput capillary fingerprinting data. Bioinformatics.

[B17] BioinforSoft (2006). FPMiner. http://bioinforsoft.com/fpminer.html.

[B18] Radulovic D, Jelveh S, Ryu S, Hamilton T, Foss E, Mao Y, Emili A (2004). Informatics platform for global proteomic profiling and biomarker discovery using liquid chromatography-tandem mass spectrometry. Molecular & cellular proteomics.

[B19] Windig W, Phalp J, Payne AW (1996). A Noise and Background Reduction Method for Component Detection in Liquid Chromatography/Mass Spectrometry. Analytical Chemistry.

[B20] Nelson W, Bharti A, Butler E, Wei F, Fuks G, Kim H, Wing R, Messing J, Soderlund C (2005). Whole-Genome Validation of High-Information-Content Fingerprinting. Plant Physiology.

[B21] Moroldo M, Paillard S, Marconi R, Fabrice L, Canaguier A, Cruaud C, Berardinis VD, Guichard C, Brunaud V, Clainche IL, Scalabrin S, Testolin R, Gaspero GD, Morgante M, Adam-Blondon AF (2008). A physical map of the heterozygous grapevine 'Cabernet Sauvignon' allows mapping candidate genes for disease resistance. BMC Plant Biology.

[B22] Jaillon O, Aury JM, Noel B, Policriti A, Clepet C, Casagrande A, Choisne N, Aubourg S, Vitulo N, Jubin C, Vezzi A, Legeai F, Hugueney P, Dasilva C, Horner D, Mica E, Jublot D, Poulain J, Bruyère C, Billault A, Segurens B, Gouyvenoux M, Ugarte E, Cattonaro F, Anthouard V, Vico V, Fabbro CD, Alaux M, Gaspero GD, Dumas V, Felice N, Paillard S, Juman I, Moroldo M, Scalabrin S, Canaguier A, Clainche IL, Malacrida G, Durand E, Pesole G, Laucou V, Chatelet P, Merdinoglu D, Delledonne M, Pezzotti M, Lecharny A, Scarpelli C, Artiguenave F, Pè M, Valle G, Morgante M, Caboche M, Adam-Blondon AF, Weissenbach J, Quétier F, Wincker P (2007). The grapevine genome sequence suggests ancestral hexaploidization in major angiosperm phyla. Nature.

