# Supplementary material for: Automated FingerPrint Background removal: FPB
Source: BMC Bioinformatics. 2009 Apr 30;10:127. doi: 10.1186/1471-2105-10-127 (PMC2689866; doi:10.1186/1471-2105-10-127)

Particular cases of peaks to be removed

Partially digested fragments caused by insufficient enzyme concentration (laboratory error) and star activity by-products caused by digestion in non canonical locations (enzyme error) result in similar fingerprints with fragments that tend to exhibit an intermediate signal intensity, between true and other background peaks. In accordance with the safety principle both cases are rejected.

Below, we present a chromatogram likely containing either partial digestions or star activity by-products: at about 1200 RFUs (y axis) there is a high number of peaks while higher (likely true) peaks show double height. In the project where this fingerprint was produced, lots of similar profiles were found in a single dye only; therefore these particular cases are likely to be related to star activity (which normally varies among different enzymes).

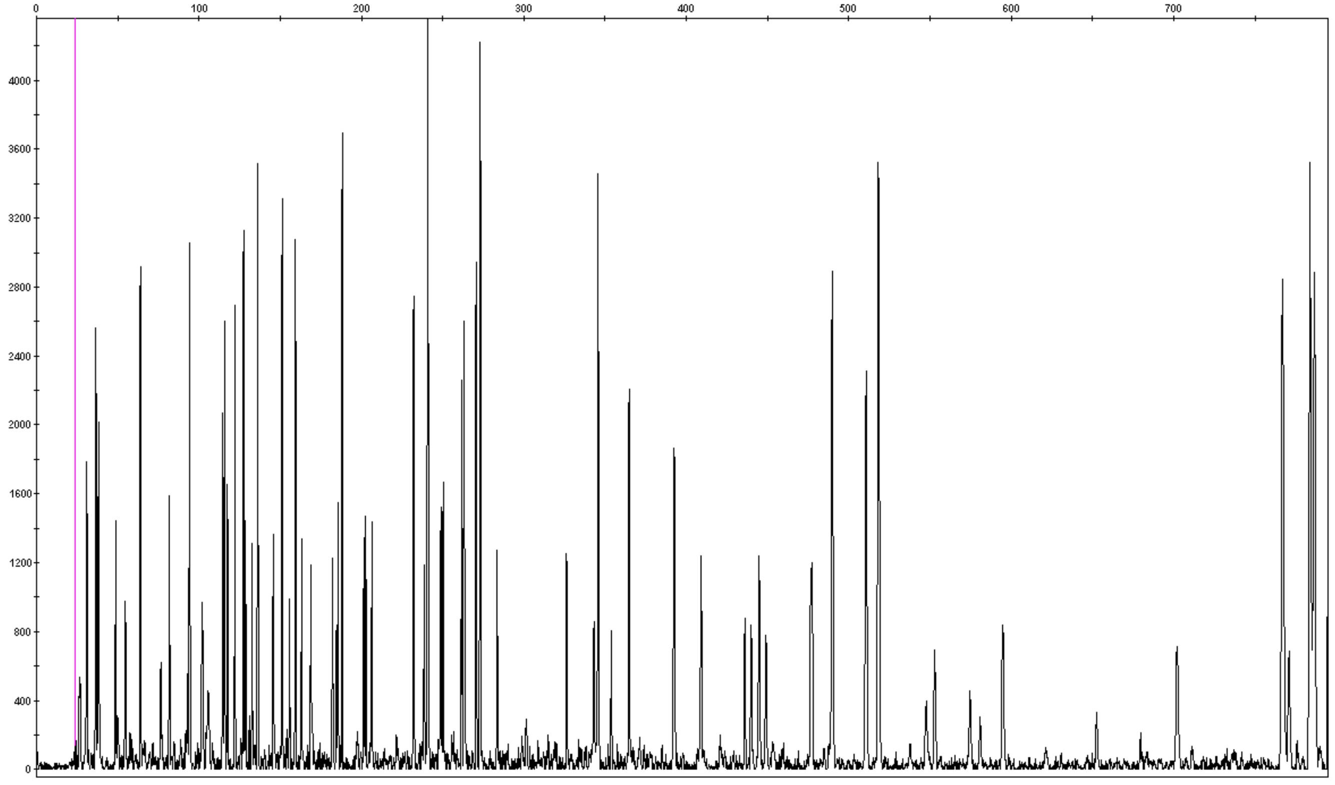

Below is shown a picture of a chromatogram for an empty well. It still does produce some peaks. That is the “machine” background.

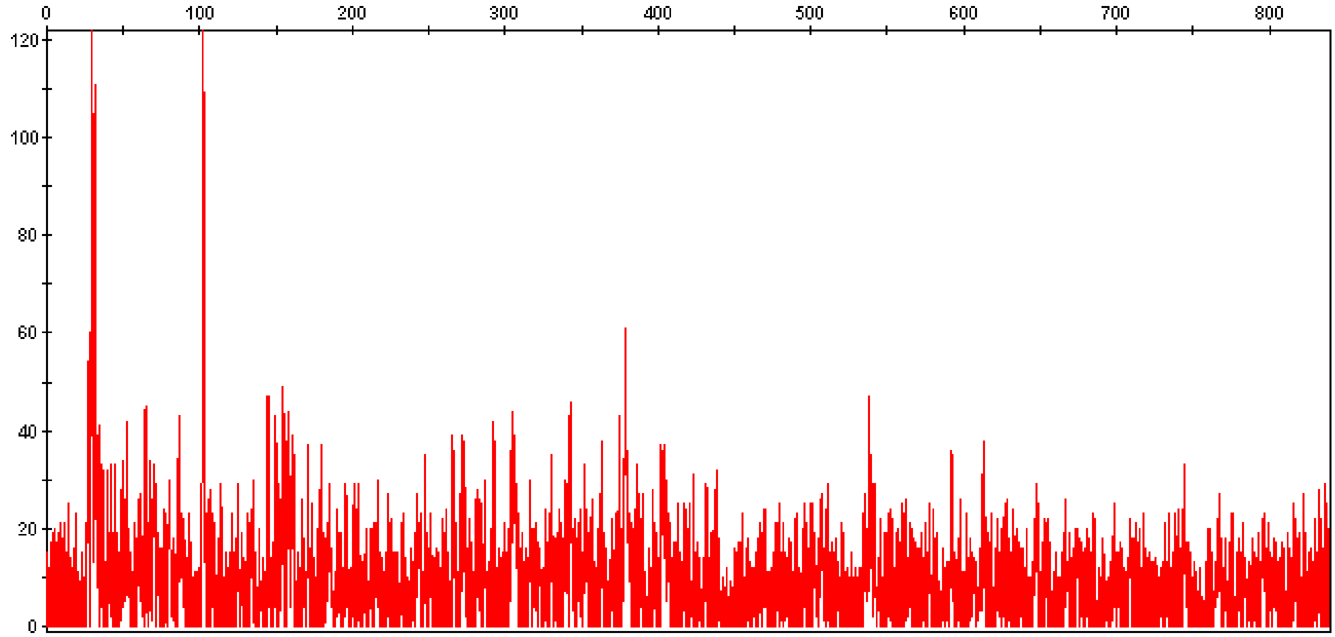

Below is shown a picture of a chromatogram with high background, mainly caused by *E. coli* peaks hardly distinguishable from true peaks.

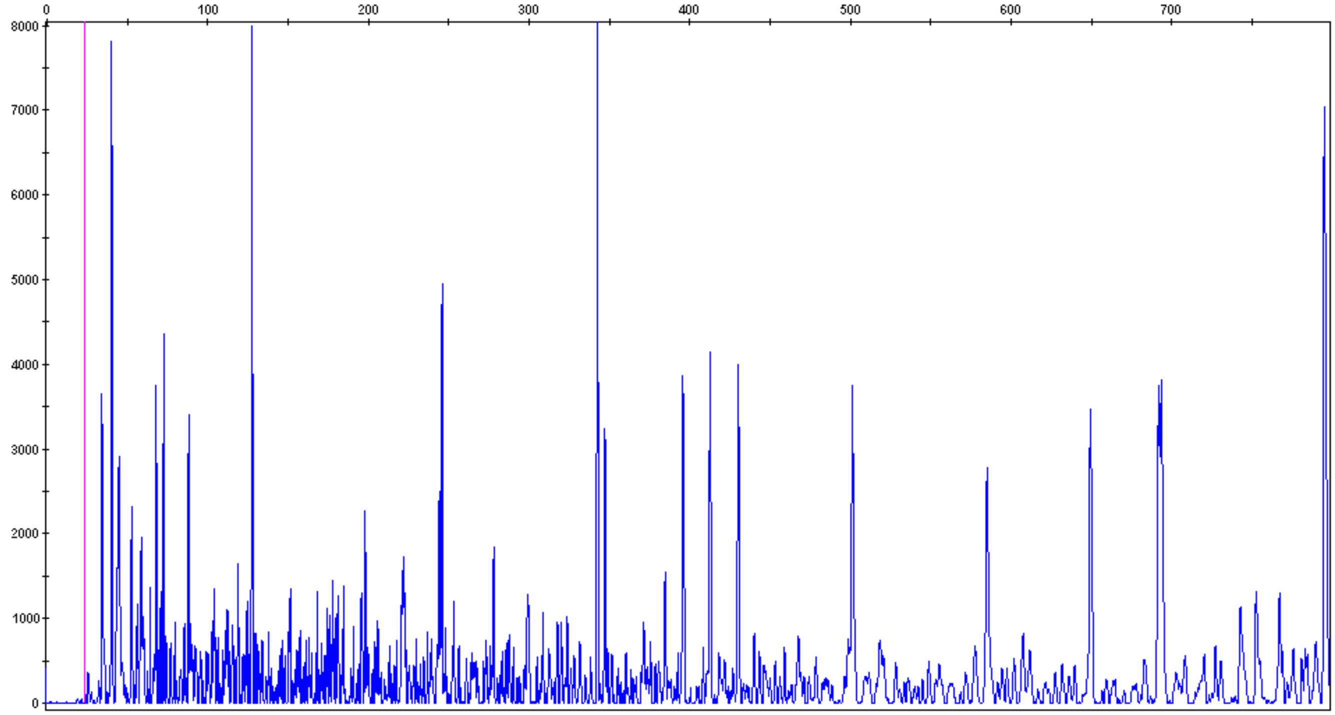

Supplement: Additional file 1 — Particular cases of peaks to be removed. Partially digested fragments, star activity by-products, "machine" background, and E. coli peaks present particular features and need to be removed accordingly. [file 1471-2105-10-127-S1.pdf]
