# Supplementary material for: Automated FingerPrint Background removal: FPB
Source: BMC Bioinformatics. 2009 Apr 30;10:127. doi: 10.1186/1471-2105-10-127 (PMC2689866; doi:10.1186/1471-2105-10-127)
Supplement: Additional file 2 — Distribution of true peaks vs background peaks. True and background peaks distribute differently from clone to clone and from dye to dye. Therefore, different scenarios may arise and particular care in background removal should be used. [file 1471-2105-10-127-S2.pdf]

Distribution of true peaks vs background peaks

We present here the distribution of the ratio of true peaks (at least putative since they are true peaks according to FPB) versus background peaks (data relative to table 1, divided per dye, after preprocessing fragments out of size standard range, vector bands, and wide area peaks). Ratios are relative to 74,766 clones of the wheat 3B chromosome project (to be precise 74,766 are obtained eliminating 7,410 that did not pass the size standard analysis in GeneMapper from a total of 82,176). 1,207 clones were initially discarded by FPB (they should fall in cases d) and e)).

Ratios are plotted for each dye/enzyme and grouped in 500 bins of size 0.2. We limited our analysis to ratios lower than 100 (only very few cases go beyond it). It is clear that most of fingerprints considered are from cases a) (we define them as those with ratio>20) and b) (we define them as those with ratio between 5 and 20) but a part is still from case c) (we define them as those with ratio<5) and should be treated correctly otherwise clones containing spurious artifactual bands are provided to FPC.

Notice however that the quality of fingerprints is strongly dependent on the quality of the BAC DNA minipreps and specifically on the amount of leftover E. coli genomic DNA as well as on the presence of other contaminants. Since different procedures for the extraction of BAC DNA are used in different laboratories, the fingerprint quality will vary accordingly. In addition to this, there are always differences among the different dyes/enzymes and this is mainly due to the enzymes themselves as can be seen from the figure hereunder.

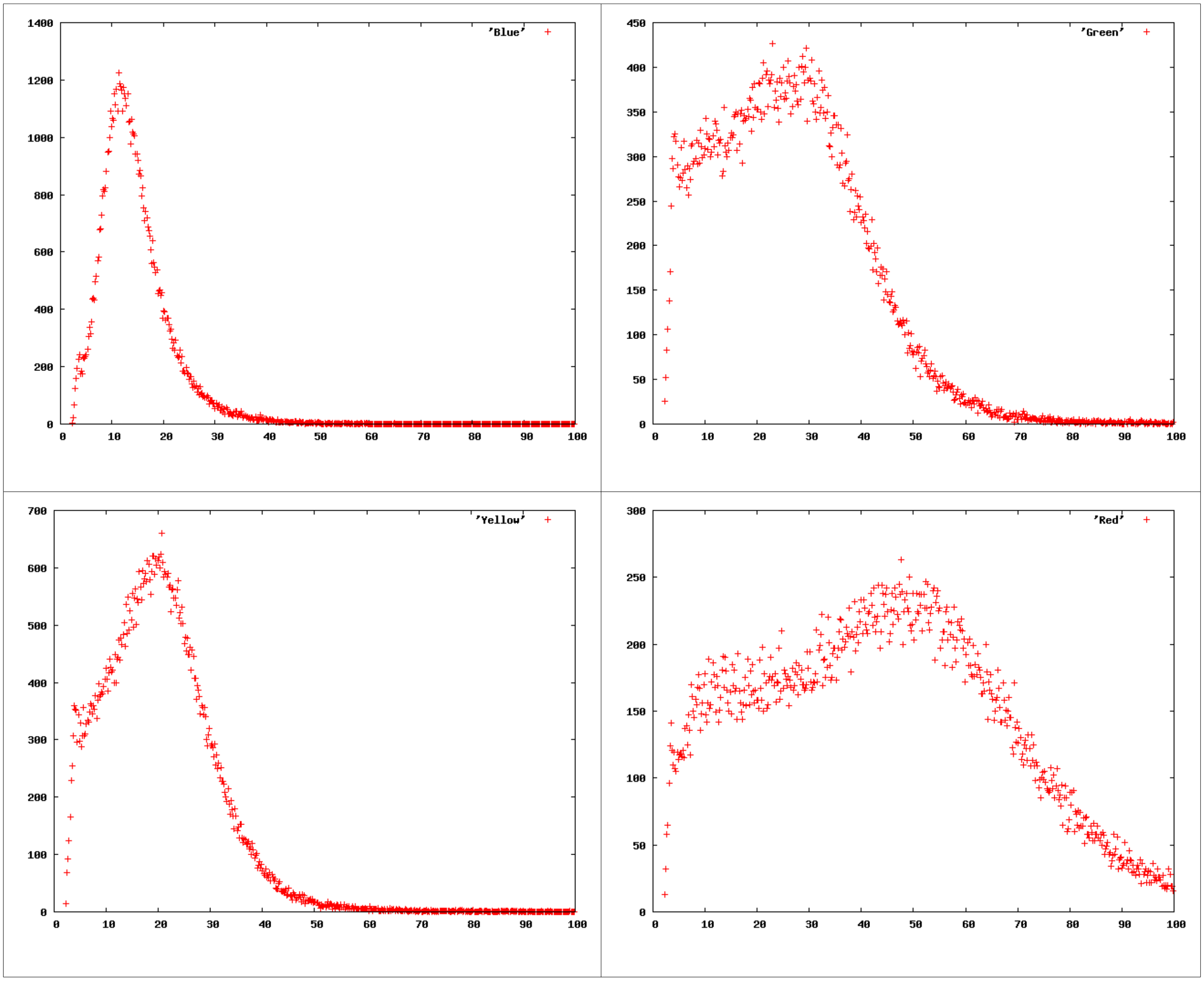
'Blue'
'Green'
'Yellow'
'Red'
